# Effect of *Allium cepa* loaded polyacrylonitrile and polyvinyl pyrrolidone nanofibers on *Candida albicans* growth and the expression of *CDR1* and *CDR2* genes

**DOI:** 10.18502/cmm.7.4.8408

**Published:** 2021-12

**Authors:** Azam Nademi, Masoomeh Shams-Ghahfarokhi, Mehdi Razzaghi-Abyaneh

**Affiliations:** 1 Department of Mycology, Faculty of Medical Sciences, Tarbiat Modares University, Tehran, Iran; 2 Department of Mycology, Pasteur Institute of Iran, Tehran, Iran

**Keywords:** *Allium cepa*, *Candida albicans*, *CDR1*/*CDR2*, Gene expression, PAN/PVP nanofibers

## Abstract

**Background and Purpose:**

This study aimed to assess the effect of *Allium cepa* ethanolic extract (ACE) loaded polyacrylonitrile (PAN) and polyvinyl pyrrolidone (PVP) nanofibers on *Candida albicans* (*C. albicans*) *CDR1* and *CDR2* genes expression.

**Materials and Methods:**

The minimum inhibitory concentrations (MICs) of ACE against *C. albicans* ATCC 10231 and clinical fluconazole (FLC)-resistant *C. albicans* PFCC 93-902 were determined using the Clinical and Laboratory Standards Institute (CLSI) protocol (M27-Ed4) at a concentration range of 45.3-5800 µg/ml. The nanofibers containing ACE (60 wt%) were fabricated using the electrospinning technique. The expression of the *CDR1* and *CDR2* genes was studied in the fungus exposed to ACE-loaded nanofibers and 0.5×MIC concentration of FLC using the real-time polymerase chain reaction.

**Results:**

MIC_50_ and MIC_90_ of ACE against FLC-resistant *C. albicans* were 725 and 1450 μg/mL, respectively. The expression of *CDR1* (4.5-fold) and *CDR2* (6.3-fold) were down-regulated after the exposure of FLC-resistant *C. albicans* to ACE-loaded nanofibers (*P*<0.05). Furthermore, the expression of *CDR1* (2.8-fold) and *CDR2* (3.2-fold) were up-regulated in FLC-treated *C. albicans* (*P*<0.05).

**Conclusion:**

The results revealed that nanofibers containing ACE interact with drug-resistant genes expressed in *C. albicans*. Further studies are recommended to investigate the mode of action and other biological activities of ACE-loaded nanofibers against *C. albicans* and other pathogenic fungi.

## Introduction

*Candida albicans* (*C. albicans*) is a widespread nosocomial pathogen that causes a variety of diseases from superficial skin and mucosal infections to life-threatening systemic infections, specifically in immunocompromised patients. Despite the rising occurrence of candidiasis, there are just a few antifungal drugs available to treat this important cosmopolitan fungal infection. Furthermore, the increased resistance of *Candida* species to various antifungal drugs has raised serious concerns and is an additional obstacle to therapy [ 1 , 2 ]. Fluconazole (FLC), an azole drug, is one of the most generally used antifungal drugs for mucosal and superficial candidiasis. *Candida* spp. are resistant to azoles, especially *C. albicans*, and have been widely documented and investigated [ 3 ]. The action of some efflux pumps reduces the intracellular azole concentration, which is the most typically documented resistant mechanism. Increased expression of *CDR1* and *CDR2* genes in *C. albicans*, which encode efflux pumps, reduces azole accumulation [ 4 ]. This indicates the need for the discovery of novel antifungal drugs. *Allium cepa* L. (*A. cepa*, onion) belongs to the Liliaceae family and has a diverse variety of species. *A. cepa* has been regarded as a powerful antimicrobial agent that can help fight infections. Many bacteria, fungi, and viruses have been reported to be susceptible to A. cepa extracts in various solvents. Sulfur compounds have been discovered to be the most effective antimicrobial agents found in *A. cepa* [ 5 - 7 ] .Electrospinning is a simple and versatile process that involves creating ultrathin fibers from a suspended drop of a polymer solution or melt using a high electric field [ 8 ]. Nanofibrous architectures created by electrospinning plant extracts and other materials have attracted a lot of research attention in the last ten years. The decreased drug toxicity, drug stability, antimicrobial, anti-inflammatory, and anti-oxidant properties of the resultant structures make them suitable for biomedical applications [ 9 , 10 ]. 

The present study aimed to investigate the effect of *Allium cepa* ethanolic extract (ACE) loaded polyacrylonitrile and polyvinyl pyrrolidone nanofibers on *C. albicans* *CDR1* and *CDR2* genes expression

## Materials and Methods

### Fungal strains and culture condition

In the present study, a FLC-susceptible *C. albicans* ATCC 10231 and a clinical FLC-resistant *C. albicans* PFCC 93-902 were obtained from the Pathogenic Fungi Culture Collection of the Pasteur Institute of Iran, Tehran, Iran, were examined. *C. albicans* strains were kept as a frozen stock in glycerol at 80 ºC. Throughout the investigation, fresh fungal cultures were generated by sub-culturing on Sabouraud dextrose agar (SDA, Merck, Germany) at 35°C for 24 h. To make the cell suspension, one colony from the SDA cultures was taken and re-suspended in Sabouraud dextrose broth (SDB, Merck, Germany) at a concentration of 1×10^6^ cells/ml [ 2 ]. 

### Preparation of Allium cepa ethanolic extract

Extraction was performed according to Musavinasab-Mobarakeh et al. with slight modifications [ 11 ]. Briefly, 1000 g of *A. cepa* (yellow onion) bulbs were blended in a mixer and dried in a freeze-dryer (Christ, Germany). To make *A. cepa* ethanolic extract, 80 g of the dried powder was combined with 800 ml of ethanol and sonicated afterward. The extracts were filtered using Whatman No. 1 filter paper after incubation at room temperature for 3 days on a shaker. Ethanol was evaporated at 40°C from the extract by a rotary evaporator.

### Fabrication of Allium cepa-loaded nanofibers

The PVP (50% w/v, Merck, Germany) and PAN polymer powders (15% w/v, Isfahan Polymer Co., Iran) were dissolved in 1 mL of 70% ethanol and dimethyl sulfoxide (Sigma-Aldrich, USA), respectively. To prepare ACE-loaded nanofibers, ACE was added to a rate equal to 40, 50, 60, and 70% of the polymer (s) weight (40, 50, 60, and 70 wt%) to electrospinning solutions. A syringe (1 mL) was used for electrospinning solution injection. The needle tip was 15 cm away from the drum and injected at a rate of 0.3 ml/h, while the needle was exposed to 10-15 kV voltages from a high-voltage power supply. Electrospun nanofibers were gathered on a 25×15 cm^2^ aluminum foil wrapped around the rotating collector [ 10 ]. 

### Characterization of nanofibers by scanning electron microscopy

A small section of the prepared electrospinning PAN/PVP solution containing 40, 50, and 60 wt% of ACE was coated with gold before imaging with scanning electron microscopy (SEM). The morphological characteristic and diameter of the nanofiber mats were determined using SEM (FEI NOVA Nano SEM 450, Netherlands) at 10 kV, followed by an optical magnification of 50,000x. The mean diameter of nanofibers (n=60) was measured using Image Analysis Software (Image J, National Institute of Health, USA) [ 10 ]. 

### Antifungal susceptibility testing

The broth microdilution reference method was used to establish minimum inhibitory concentrations (MICs), as specified by CLSI guidelines M27-Ed4 [ 12 ]. For the CLSI microdilution trays, reagent-grade powders of FLC (Pfizer Central Research, Sandwich, Kent, UK) were purchased from the respective manufacturers. To obtain final concentrations of 45.3 to 5800 µg/mL, the ACE was prepared in two-fold serial dilutions in RPMI-1640 (Sigma Aldrich, USA) in a microplate. From a stock solution of FLC, successive two-fold concentrations of 0.0313-64 µg/mL were produced as drug control. Each well of a 96-well microplate was then filled with a 100 µL cell suspension of *C. albicans* (0.5-2.5×10^3^ CFU/mL) produced in RPMI- plus MOPS (3-(N-morpholino) propane sulfonic acid) medium. Microplates were incubated for 24 h at 35°C. The RPMI medium with fungal cells was employed as a drug-free control. The CLSI M27-Ed4 was used to interpret the MIC values. Assay for minimum fungicidal concentration (MFC) was conducted by taking 50 µL of the cultures from any wells with no obvious fungal growths and plating them on SDA plates. The amount of fungal growth was determined subsequently. MFC was defined as the lowest concentration required to kill at least 99.9% of the main inoculums after incubation at 35°C for 24 h.

### RNA extraction and quantitative Real-Time RT-PCR assay

Total RNA was extracted from ACE 60%-loaded nanofibers and FLC-treated *C. albicans* strains at 0.5×MIC concentration, compared to controls (non-treated *C. albicans* strains). The RNAX plus kit (Sina clone, Iran) was used to extract RNA from *C. albicans* strains following the manufacturer’s instructions. Spectrophotometric measurements and run-on agarose gel were used to quantify RNA concentrations and purity (Figure S1). Following the technique, first-strand cDNA was synthesized from 1000 ng of RNA using a cDNA reverse transcription kit (Vivantis, Malaysia). The primers sets included *CDR1* (F5ˊ-CTTAGTCAAACCACTGGATCG, R5ˊ-CCAAAAGTGATGAAAGGC), *CDR2* (F5ˊ-CACGTCTTTGTCGCAACAGC, R5ˊ-ATGTTGTGACTTGCAGCAGTAGC), and *ACT1* (F5ˊ-GAGTTGCTCCAGAAGAACATCCAG, F5ˊ-TGAGTAACACCATCACCAGAATCC) [ 13 , 14 ]. Product quality RT-PCR was performed before Real-time PCR (Figure S2). 

RT-qPCR was performed by Corbett Rotor-Gene 6000 real-time PCR cycler (Qiagen Corbett, Hilden, Germany) with an initial denaturation step at 95°C for 4 min, 40 cycles of 95°C for 15 s, 54°C for 15 s, and 72°C for 45 s. Negative controls were provided in each run. All data were normalized using the internal reference gene *ACT1* as a housekeeping gene. The relative target-gene expression was calculated as a fold change of 2^-ΔCT^ value, in which ΔCT=CT (target gene) – CT (internal reference genes). The experiments were carried out in sets of three. The results were calculated using GraphPad PRISM 9 (GraphPad Prism Software Inc., USA). A one-way ANOVA was used for the statistical analysis, and a *P-value* less than 0.05 (*P*<0.05) was considered statistically significant.

## Results

### Morphology and average diameter of nanofibers

**Figure 1:**
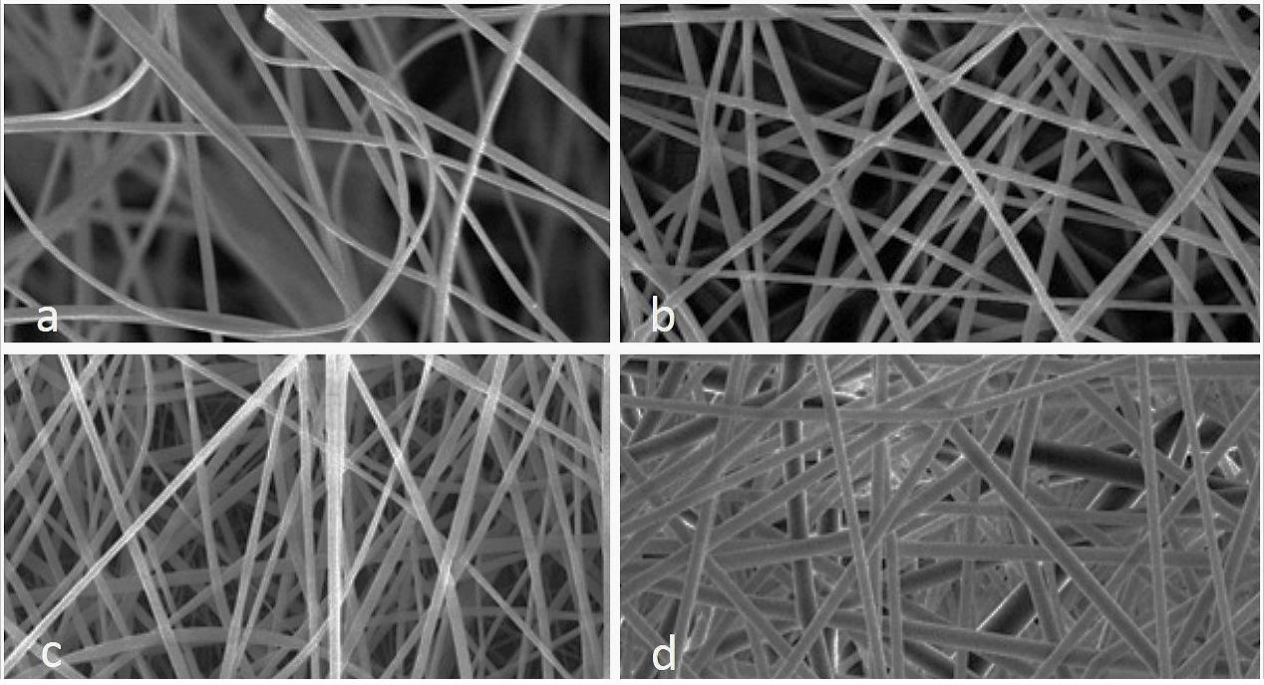
SEM images of PAN/PVP nanofibers loaded with ACE a) 0%, b) 40%, c) 50%, and d) 60 %

The morphologic characteristics and average diameter of PAN/PVP nanofibers loaded with different concentrations of ACE (40%, 50%, and 60%) were determined by SEM (Figure 1). The maximum percentage in terms of electrospinning ability and possession of the most uniform fiber morphology was observed to be 60%, according to SEM images. The mean±SD diameter of synthesized ACE 60%-loaded nanofibers was 1206±30.4 nm. One-way ANOVA was applied to analyze the effect of ACE content on the mean diameter of nanofibers, and it was revealed that the increase in fiber diameter was considered statistically significant (*P*<0.05).

### Antifungal susceptibility

*In vitro* antifungal activity profiles of compounds against *C. albicans* ATCC 10231 and PFCC 93-902 are described in Table 1. Results of antifungal susceptibility testing showed that MIC and MFC of ACE against FLC-resistant *C. albicans* PFCC 93-902 were 1450 and 2900 µg/mL, respectively. MIC and MFC for *C. albicans* ATCC 10231 were 725 and 1450 µg/mL, respectively. Moreover, MICs of FLC for *C. albicans* ATCC 10231 and PFCC 93-902 strains were 0.25 and 64 µg/ml, respectively. The MFCs were two times higher than their MICs in strains of *C. albicans*.

**Table 1:** Antifungal activities of Allium cepa ethanolic extract (ACE) and Fluconazole (FLC) against C. albicans strains by the broth microdilution method

Fungal strain	Antifungal compound	MIC range	MICs (µg/mL)		MFC
			MIC_50_	MIC_90_	
*C. albicans* ATCC 10231	ACE	45.3-5800	362.5	725	1450
	FLC	0.031-64	0.125	0.25	0.5
*C. albicans* PFCC 93-902	ACE	45.3-5800	725	64	128
	FLC	0.031-64	32	1450	2900

MIC: Minimum inhibitory concentration; MFC: Minimum fungicidal concentration

### Real-time PCR assay

**Figure 2:**
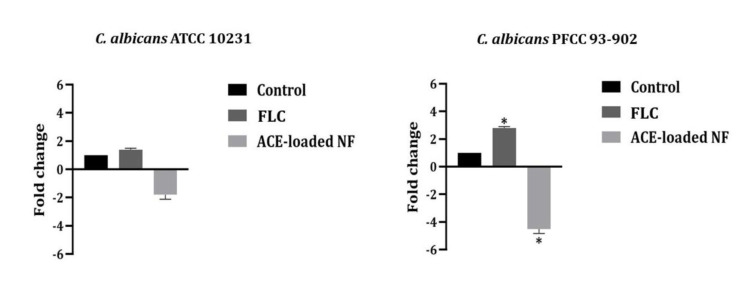
The fold changes of *CDR1* determined by qRT-PCR for ACE-loaded NF in *C. albicans* strains. *Statistically significant difference with a control. ACE: *Allium cepa* ethanolic extract; NF: Nanofiber; FLC: Fluconazole

**Figure 3:**
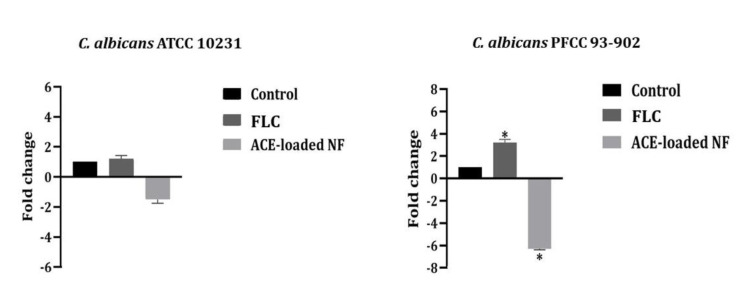
The fold changes of *CDR2* determined by qRT-PCR for ACE-loaded NF in *C. albicans* strains. *Statistically significant difference with a control. ACE: *Allium cepa* ethanolic extract; NF: Nanofiber ; FLC: Fluconazole

The purity of extracted RNA in treated and non-treated *C. albicans* strains was analyzed using agarose gel electrophoresis (1%). RT-PCR products in different PCR conditions were compared in ACE-, FLC-treated and non-treated *C. albicans* strains. The analysis of the expression of *CDR1* and *CDR2* genes using one-way ANOVA revealed that the of expression *these* genes were significantly down-regulated to 4.5 and 6.3-folds in the clinical strain of *C. albicans* PFCC 93-902 (FLC-resistant) after treatment with ACE-loaded nanofibers (*P*<0.05) (Figures 2 and 3). However, the expression of *CDR1* and *CDR2* was up-regulated to 2.8 and 3.2 folds in *C. albicans* PFCC 93-902 strain when treated with fluconazole (*P*<0.05) (Figure 2 and 3). Although, a difference was observed between *CDR1* and *CDR2* expression in ACE-loaded nanofibers and FLC–treated *C. albicans* ATCC 10231, this difference was not statistically significant, compared to non-treated control (*P*>0.05).

## Discussion

Many bioactive ingredients and herbal compounds, which have traditionally been utilized to promote wound healing, are found in plants. Many plant extracts or purified main fractions used in herbal medicine have been shown to have therapeutic effects similar to less toxic pharmaceuticals [ 15 ]. *A. cepa* has been considered a powerful antibiotic agent to combat infectious diseases. Many bacterial, fungal, and viral species were found to be sensitive to *A. cepa* solvents [ 16 , 17 ]. Several studies have confirmed that *A. cepa* contains antifungal compounds, such as organo-sulfur derivative components with antifungal activity against important pathogenic fungi, including yeasts and filamentous fungi [ 18 , 19 ]. and Korukluoglu found that the ethyl alcohol extract of *A. cepa* effectively inhibited the growth of *Aspergillus niger* (MFC=275 mg/mL) [ 19 ]. Susceptibility to crude ethanol extracts fresh *A. cepa* and aqueous *A. cepa* extracts (50% concentration) for *C. albicans* have been reported as well [ 20 , 21 ]. Shams-Ghahfarokhi et al. showed that MICs of aqueous extracts of fresh *A. cepa* were 4.522 mg/ml and 8.062 mg/ml for *C. albicans* and *Malassezia furfur*, respectively [ 22 ]. Gomaa et al. showed that *A. cepa* extract biosynthesized silver nanoparticles (AgNPs) had the highest MIC of 10 mg/mL against C. albicans ATCC 70014 among tested microorganisms [ 23 ]. 

In this study, *in vitro* antifungal activity of ACE was compared to FLC as a clinically effective antifungal agent. The obtained results showed that ACE inhibited the clinical FLC-resistant strain growth by 50% at 725 μg/mL, while it inhibited the fungal growth completely at the concentration of 2900 μg/mL. ACE (MIC=2000 μg/mL) has been shown to effectively inhibit *Cryptococcus neoformans* growth and pathogenicity through influencing cell membrane ergosterol concentration, laccase activity, melanin generation, and *LAC1* gene expression [ 11 ]. 

Due to various properties, such as biocompatibility, controlled drug release efficiency, and tailoring ability, nanofiber scaffolds with loaded pharmaceuticals have recently attracted interest for the creation of wound dressings, particularly in skin tissue engineering [ 24 ]. Several studies demonstrated that sertaconazole incorporated polyurethane/polyvinylpyrrolidone /silk nanofibers, PAN loaded with eugenol, and polycaprolactone/polystyrene nanofibrous mats containing chamomile were fungistatic against *C. albicans* with excellent biocompatibility, suggesting that they could be used as a scaffold in the treatment of fungal infections [ 25 - 27 ]. 

Little is known regarding the mode of antifungal action of nanofibers containing drugs. To the best of our knowledge, this is the first study on the effect of ACE-loaded nanofibers on *CDR1* and *CDR2* genes expression in *C. albicans*. To date, a number of azole-resistant genes (e.g., CDR1, CDR2, MDR1, ERG3, ERG6, ERG11, ERG9, RTA2, and NAG2) have been identified. Each of these genes develops antifungal drug resistance in the organism through various molecular processes [ 28 ]. The *CDR* gene family in *C. albicans* includes a number of genes of which only the role of *CDR1* and *CDR2* has been documented in relation to fluconazole resistance in different fungi. These genes have been shown to be overexpressed in *C. albicans* azole-resistant isolates. It has been claimed that overexpression of efflux pumps encoded by the *CDR1*, *CDR2*, and multidrug-resistant 1 (*MDR1*) genes is one of the most frequent mechanisms of fluconazole resistance in *Candida* species. Cdr1p and Cdr2p, plasma membrane proteins produced by the ABC transporter genes *CDR1* and *CDR2*, are significant factors affecting FLC-resistant in Candida [ 29 - 32 ]. *CDR1* and *CDR2* overexpression has been associated with fluconazole resistance isolates in *C. albicans* and could not be determined in the fluconazole susceptible isolates [ 29 , 31 , 33 ]. Despite their considerable sequence similarity, Cdr1p contributes more significantly to FLC resistance in *C. albicans* than Cdr2p. On the other hand, up-regulation of multidrug efflux pump controlled by Cdr1p, and Cdr2p belonging to ATP-binding cassette superfamily (APC transporter) were implicated in most fluconazole-resistant *C. albicans* strains as FLC was a substrate for CDR1, CDR2 [ 31 , 33 ].

In the present study, *CDR1*/*CDR2* genes expression in ACE-loaded nanofibers treated *C. albicans* FLC-resistant strain down-regulated to 4.5 and 6.3-folds, respectively. The *CDR2* expression was (6.3-fold) more effectively decreased than *CDR1* (4.5-fold) in FLC-resistant *C. albicans*; however, the expression of *CDR1* and *CDR2* were up-regulated to 2.8 and 3.2-folds in FLC-resistant *C. albicans* strain after treatment with fluconazole. Based on current literature, in both *C. albicans* FLC-resistant and FLC-susceptible strains, expression of *CDR1* and *CDR2* genes is increased in FLC-treated samples, while the effect of some substances, possibly with different mechanisms of FLC function on gene expression, leads to their down-regulation [ 21 , 28 ]. In this study, the results showed that the expression of *CDR1* and *CDR2* genes decreased and down-regulated in FLC-resistant *C. albicans* exposed to ACE-loaded nanofibers, while it was up-regulated in the fungus exposed to FLC. This may be due to the higher antifungal activity of FLC compared to nanoformulated ACE and further indicates non-predictable behavior of gene expression in the presence of unknown complex substances, such as ACE.

It has been shown that herbal products and their active constituents in combination with antifungal drugs could decrease the drug resistance of *Candida* species through the suppression of *CDR1* and expression of *MDR1* genes which result in increased intracellular concentration of antifungal drugs and, in turn, the effectiveness of those drugs against resistant *Candida* strains [ 34 ]. 

In this study, ACE-loaded nanofiber reduced the activity of the transporter-mediated efflux pump, especially by the decreased expression of *CDR1* and *CDR2*. The down-regulation of these genes indicates that ACE-loaded nanofibers can reduce the resistance of *C. albicans* to an antifungal drug by decreasing the expression of the drug-related genes with different mechanisms, compared to the conventional antifungal agents.

## Conclusion

In conclusion, the obtained results showed that nanoformulated ACE effectively inhibited the growth of FLC-susceptible and resistant *C. albicans* strains. Nanofibers containing ACE fabricated with electrospinning significantly suppressed the expression of *CDR1* and *CDR2* genes, which encode efflux pumps, in FLC-resistant *C. albicans*. Taken together, these results indicate that the nanoformulated ACE can be considered as a novel nanofiber that may be effective in the treatment of skin and mucosal candidiasis.

## Acknowledgement

Research reported in this publication was supported by Elite Researcher Grant Committee under the award number (grant no. 963366) from the National Institute for Medical Research Development (NIMAD), Tehran, Iran. Financial supports from the Research Deputy of Tarbiat Modares University are also acknowledged.

## Authors’ contributiontitle

A.N. and M.S.G. conceptualized the study. A.N., M.S.G., and M.R.A. performed data collection and formal analysis. A.N. and M.S.G. undertook the required investigation. A.N. and M.S.G. selected the methodology and administered the project. M.S.G. supervised the study. M.S.G. and M.R.A. validated the data. A.N. wrote the original draft. A.N., M.S.G., and M.R.A. wrote, reviewed, and edited the final draft.

## Conflict of Interest

The authors declare no conflict of interest regarding the publication of this study. 

## Financial disclosure

The authors disclose no relevant financial interests regarding this study. 
